# Clinical Characteristics and Outcomes of Pediatric Vitamin C Deficiency

**DOI:** 10.3390/nu17233755

**Published:** 2025-11-29

**Authors:** Thanaporn Trangkanont, Maneerat Puwanant, Thirachit Chotsampancharoen

**Affiliations:** 1 Nutrition Division, Department of Pediatrics, Faculty of Medicine, Prince of Songkla University, Hat Yai 90110, Thailand; 2 Hematology and Oncology Division, Department of Pediatrics, Faculty of Medicine, Prince of Songkla University, Hat Yai 90110, Thailand

**Keywords:** children, pediatric, vitamin C deficiency, scurvy, ascorbic acid

## Abstract

**Background:** Vitamin C deficiency remains an under-recognized condition in children, especially in Southeast Asia. This study aimed to study the clinical characteristics, dietary risk factors, and outcomes of pediatric vitamin C deficiency in a tertiary hospital in Southern Thailand. **Methods:** This retrospective study reviewed the medical records of children aged 1 to 15 years diagnosed with vitamin C deficiency from 2004 to 2024. Diagnosis was based on serum ascorbic acid levels below 0.4 mg/dL, or clinical-radiographic findings. Data collected included demographics, developmental status, dietary history, clinical presentations, radiological and laboratory results, treatment, and outcomes. **Results:** Forty-six children were diagnosed; the median age was 4.8 years, and 60% were male; developmental delay was present in 54.4%. The majority had poor dietary intake, with 73% not consuming adequate fruits and vegetables; no significant differences were observed when classified by developmental status. Common clinical signs included: limb pain (76.1%), refusal to walk (52.2%), and bleeding gums (39.1%). Radiographs showed osteopenia in 73.8% and white line of Frankel in 47.6%. Serum ascorbic acid deficiency was significantly associated with low fruit and vegetable intake and excessive milk consumption over the recommendations (*p* < 0.05). Treatment with oral vitamin C resulted in clinical improvement; although, residual symptoms persisted in some cases. **Conclusions:** As pediatric vitamin C deficiency is still of concern, this study highlights the importance of early detection in at-risk children and the critical role of detailed dietary history to identify inadequate nutrition. Prompt recognition and intervention can prevent misdiagnosis and improve clinical outcomes. Hence, strengthening parental education on nutritional intake is essential to reduce future incidences.

## 1. Introduction

Ascorbic acid (vitamin C) is a water-soluble vitamin that cannot be synthesized by the human body due to the absence of the enzyme L-gulonolactone oxidase. The only source of vitamin C for the body is through diet [1,2]. Vitamin C plays a vital role as a cofactor for several enzymes involved in collagen synthesis, neurotransmitter production, and enhancing immune function [1].

Vitamin C deficiency is a silent issue occurring worldwide. Although, the prevalence of vitamin C deficiency in children varies, depending on different studies; wherein, in developed countries the prevalence is low (reported as 1.6% of children aged 6–11 years in the United States) [3], it is still trending as an increase over time [4]. In contrast, in low to middle income countries, the prevalence can be as high as 23% [5]. In Southeast Asia, data on vitamin C deficiency prevalence in the general pediatric population are limited. However, regional data from the Southeast Asian Nutrition Survey II (SEANUT II) revealed 66.5% of Thai children age 6 months to 12 years had inadequate dietary vitamin C intake [6]. This may be a significant risk factor for developing vitamin C deficiency.

Vitamin C deficiency can cause a variety of symptoms and signs that develop typically after 4–6 weeks of inadequate intake [1,2]. The common manifestations of vitamin C deficiency include: limb pain, refusal to walk, and bleeding gums, which can mimic other diseases leading to misdiagnosis and delayed treatment [7]. If left untreated, vitamin C deficiency can result in serious complications such as pulmonary hypertension, cardiovascular collapse, and potentially fatal outcomes [8,9].

Previous studies have identified several factors and underlying conditions that influence vitamin C status in children. Low vitamin C intake is a key determinant of vitamin C deficiency [10] and can be caused by several conditions, including food insecurity, low socioeconomic status [10], feeding difficulties, and highly selective eating behaviors, all of which are common in children with neurodevelopmental disorders [7,11,12] and behavior problems [13,14,15]. Moreover, the vitamin C content in foods varies by countries depending on cultural dietary patterns and regional cooking methods [10]. Food preparation methods at high temperatures, e.g., ultra-high temperature (UHT) milk processing, can cause the vitamin C to decompose; as vitamin C is sensitive to heat it will eventually lower the vitamin C content in food [16,17]. Vitamin C deficiency can also occur in patients with gastrointestinal malabsorption [18] and those with end-stage renal disease undergoing dialysis [19], leading to excessive vitamin C loss. In certain conditions, such as burns and critical illness, vitamin C requirements increase significantly due to its crucial role in scavenging oxidative stress and modulating the inflammatory response following injury [20,21]. This may lead to a risk of vitamin C deficiency in these patients. However, the relative significance of these factors is still uncertain, as the available data are limited and are occasionally contradictory [11,22]; additionally, the influence of dietary habits on vitamin C status still requires further investigation.

Due to limited data on the clinical characteristics and dietary risk factors of vitamin C deficiency in Southeast Asian children, this study aimed to study the clinical presentations, associated risk factors of vitamin C deficiency, and outcomes of treatment.

## 2. Materials and Methods

This retrospective study reviewed the medical records of children aged 1 to 15 years having been diagnosed with vitamin C deficiency at Songklanagarind Hospital, a major tertiary university hospital in Southern Thailand. Diagnosis was identified using ICD-10 code E54 for ascorbic acid deficiency, as extracted from the hospital’s database system. Data were gathered retrospectively over a 20-year period from 2004 to 2024, as available through the standardized electronic medical record system. Access to the medical records were restricted to the physicians directly involved in patient care, and all records cannot be modified after documentation. Vitamin C deficiency was confirmed either through laboratory tests measuring serum ascorbic acid levels or based on clinical and radiographic findings when blood tests were unavailable.

### 2.1. Diagnostic Criteria

Vitamin C deficiency was confirmed in patients with serum ascorbic acid levels below 0.4 mg/dL [23]. In the absence of test results, diagnosis was also made by looking at clinical features with pathognomonic radiographic findings of vitamin C deficiency, being confirmed by a pediatric radiologist [24,25,26,27]. Characteristic radiographic findings included: Wimberger’s ring (circular calcification around the epiphyseal ossification center), white line of Frankel (dense metaphyseal line), Pelken’s spur (metaphyseal bony projections), the Trümmerfeld zone (a transverse radiolucent band beneath the white line of Frankel), and evidence of subperiosteal hemorrhage [4]. These radiographic features are highly specific for vitamin C deficiency and can therefore be used for an effective diagnosis. However, they are not sufficiently sensitive so as to exclude vitamin C deficiency if these radiologic findings are absent [4]. The clinical manifestations that must be included for diagnosis are musculoskeletal manifestations such as refusal to walk, abnormal gait, limp pain, joint swelling, and scorbutic rosary as well as hematologic manifestations including bleeding gums, epistaxis, and petechiae or ecchymosis [27].

### 2.2. Data Collection

Demographic data were collected, including age, gender, underlying diseases, and developmental status. Additionally, anthropometric measurements included weight, height, and nutritional status as BMI for age z-score, according to the World Health Organizations (WHO) criteria.

The developmental status was assessed in all patients and classified according to clinical diagnosis by a pediatrician. If developmental delay was identified, a further review was performed to determine the underlying cause. Children with a primary brain insult or conditions affecting brain function, such as cerebral palsy or epilepsy, were classified by a pediatric neurologist as having neurological impairment. Such neurological impairments may cause oromotor dysfunction leading to poor feeding [28]. In contrast, patients without such conditions, or those with isolated social or language delays, were categorized as having behavioral problems by a developmental pediatrician.

The patient’s dietary history was obtained from an interview with their caregiver conducted by a nutritionist, focusing on food variety, food quantities, and food texture. Food quantities were estimated as the portion size via food models and standard household measuring tools. This was to visually compare the food consumed for accuracy of data. Food texture refers to the characteristics of food, being described as the thickness or thinness of liquid or semisolid food. Dietary intake adequacy was compared to the Thai dietary guidelines [29,30], which include five major food groups: carbohydrates, protein, vegetables, fruits, and milk. This is used to assess if dietary intakes meet the patient’s nutritional needs based on their age and gender. In terms of milk consumption, the guidelines recommend two portions of milk per day (400 mL). Non-nutritive snacks are defined as snacks that provide calories and few essential nutrients.

Clinical presentations, physical examination findings, radiological findings, and laboratory results, including other micronutrients levels, were also collected. Treatment details and outcomes were reviewed from the medical records.

### 2.3. Biochemical Assessment

Serum ascorbic acid levels were measured using high-performance liquid chromatography (HPLC) and categorized into two groups. Deficiency was defined as a level below 0.2 mg/dL, while levels between 0.2 and 0.4 mg/dL were classified as marginal deficiency [23]. Other micronutrients were assessed based on the risk of deficiency identified during dietary review such as vitamin A, vitamin B1, vitamin D as 25-hydroxyvitamin D (25(OH)D), zinc level, and iron studies. Laboratory results were interpreted according to age-specific pediatric reference intervals [31,32].

### 2.4. Statistical Analysis

Data were analyzed using R version 4.4.3. Descriptive statistics were reported as percentages, medians with interquartile ranges (IQR), and means with standard deviations (SD). The correlation between categorical variables and categorical outcomes was assessed using Fisher’s exact test or the chi-square test. The association between potential variables and vitamin C levels was evaluated using the Wilcoxon rank-sum test and the Kruskal–Wallis test. A *p*-value of <0.05 was considered statistically significant.

## 3. Results

### 3.1. Patient Characteristic

A total of 46 patients were diagnosed with vitamin C deficiency. Of these, 30 patients were confirmed based on serum ascorbic acid levels, while 16 were diagnosed through clinical and radiologic findings. The median age at diagnosis was 4.8 years (IQR: 2.6–8.4), with 60 percent of patients being male. Nutritional status was available for 42 patients: 23 patients (54.8%) had normal nutritional status, 13 patients (31%) had wasting, and 6 patients (14.2%) were obese.

Patients were categorized by developmental status and underlying conditions. Normal development was observed in 21 patients (45.6%), while 25 patients (54.4%) had developmental delays. Among those with delayed development, 11 patients had behavioral problems associated with autism spectrum disorder, and 14 patients had neurological impairment such as epilepsy or cerebral palsy. Significant differences were observed between groups; wherein, patients with neurological impairment were diagnosed at an older age with more than half being diagnosed after 5 years of age. These patients were more likely to present with wasting compared to those with normal development and those with behavioral problems, with statistical significance (*p*-values = 0.018 and <0.001, respectively). The details of baseline characteristics are shown in Table 1.

### 3.2. Dietary Review

All patients had inappropriate food habits in terms of either food texture or quantity. Altogether, 15 out of 46 patients (32.6%) had inappropriate food textures, with 36 out of 45 patients (80%) having inadequate carbohydrate intake. Additionally, 31 out of 42 patients (73.8%) had inadequate protein intake, 33 out of 43 patients (76.7%) reported no consumption of vegetables, and 33 of 45 patients (73.3%) reported no consumption of fruits. Among those that did consume vegetables and fruits, the average frequency of consumption was 3.2 and 2.2 days per week, respectively.

In total, 18 of 21 patients (85.7%) consumed non-nutritive snacks; 38 out of 46 patients (79.5%) consumed milk, predominantly in the form of ultra-high temperature (UHT) processed milk. The median milk intake was 1040 mL/day (IQR: 360–1619), with 7 out of 38 patients (18%) consuming more than 2000 mL/day, which significantly exceeds the recommendations.

When considering developmental status subgroups, patients with neurological impairment showed a significantly higher prevalence of inappropriate food texture for their age (57.1%) compared to 28.6% in patients with normal development and 9.1% in patients with behavioral problems (*p* = 0.042). Patients with neurological impairment also had significantly higher rates of inadequate rice consumption (*p* = 0.037) whereas patients with normal development had a higher rate of inadequate meat intake (*p* = 0.04). All subgroups reported a high prevalence of patients that did not consume fruits. This accounted for 66.7% in the normal development group, 76.9% in the neurological impairment group, and 81.8% in the behavioral problem group. Similarly, a high prevalence of patients did not consume vegetables: 84.2% in the normal development group, 57.1% in the neurological impairment group, and 90% in the behavioral problem group. However, there were no significant differences between groups (Table 2).

### 3.3. Clinical Presentations

Clinical manifestations revealed limb pain (76.1%), abnormal gait (65.2%), refusal to walk (52.2%), joint swelling (28.3%), gum bleeding (39.1%), gum hypertrophy (34.8%), petechiae and ecchymosis (19.6%), perifollicular hemorrhage (8.7%), and scorbutic rosary (6.5%).

Based on the severity of the vitamin C deficiency, patients with deficiency (serum ascorbic acid <0.2 mg/dL) more frequently presented with refusal to walk compared to those with marginal vitamin C deficiency (serum ascorbic acid 0.2–0.4 mg/dL) (*p* < 0.05).

### 3.4. Radiological Study

**Figure 1:**
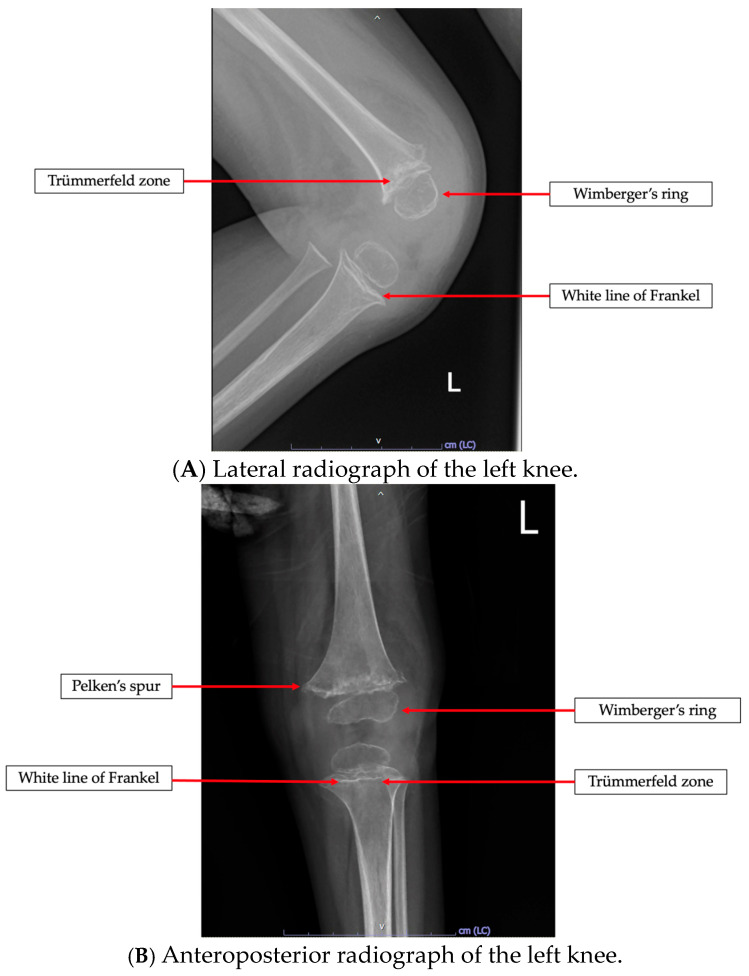
Radiologic findings in a previously healthy 2-year-old girl having presented with refusal to walk. The images demonstrate pathognomonic signs of vitamin C deficiency, including Wimberger’s ring, white line of Frankel, and Trümmerfeld zone in (**A**,**B**). Pelken’s spur is specifically highlighted in (**B**).

Among the 42 patients having undergone radiologic investigations, 34 patients (80.9%) had abnormal X-ray findings. The most common radiologic finding was osteopenia, which was observed in 31 patients (73.8%), followed by the white line of Frankel in 20 patients (47.6%), Wimberger’s ring in 16 patients (38.1%), and Trümmerfeld zone in 15 patients (35.7%). Pelken’s spur was seen in eight patients (19%), and subperiosteal hemorrhage was noted in four patients (9.5%). An example of a pathognomonic radiologic finding of this present study is a 2-year-old girl having presented with refusal to walk, as shown in Figure 1.

### 3.5. Laboratory Findings

Serum ascorbic acid levels were assessed in 30 patients, with a median level of 0.021 mg/dL (IQR: 0.001–0.110 mg/dL). Of these, 24 patients (80%) had deficiency (<0.2 mg/dL) and 6 patients (20%) had marginal deficiency (0.2–0.4 mg/dL). Among the deficiency group, 11 out of 24 patients (45.8%) had serum ascorbic acid levels lower than 0.010 mg/dL.

Additionally, other nutrient deficiencies were observed, including vitamin A deficiency in 3 out of 10 patients (30%), vitamin B1 deficiency in 1 out of 7 patients (14.3%), and vitamin D deficiency in 10 out of 16 patients (62.5%). Furthermore, zinc deficiency was found in 16 out of 29 patients (55.2%), and iron deficiency anemia was identified in 23 out of 41 patients (56%).

### 3.6. Factors Associated with Serum Ascorbic Acid Levels

**Figure 2:**
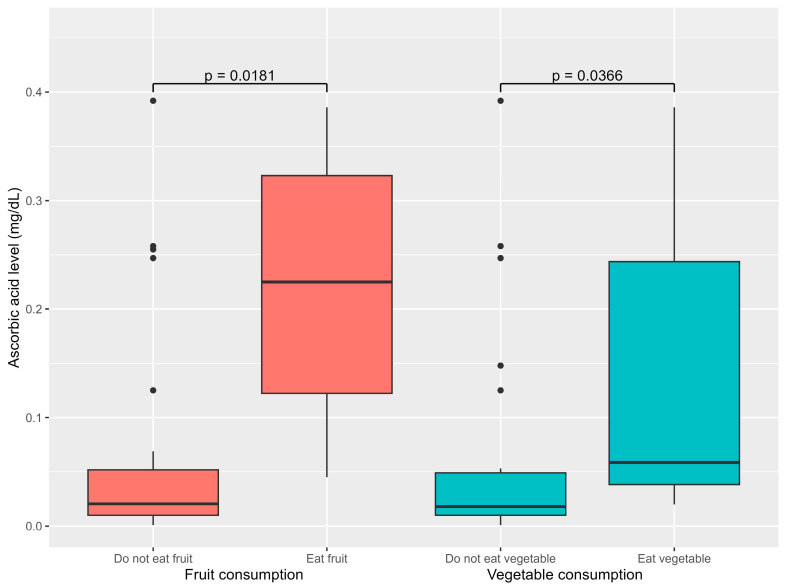
Association between dietary factor and ascorbic acid levels.

Consumption of fruits, vegetables, and milk was associated with ascorbic acid levels. Patients that consumed vegetables had higher median ascorbic acid levels of 0.059 mg/dL (0.038–0.244) compared to 0.018 mg/dL (0.010–0.049) for those that did not (*p* = 0.039). Similarly, patients that consumed fruits had significantly higher median ascorbic acid levels of 0.225 mg/dL (0.122–0.323) than those who did not. The median ascorbic acid level was 0.021 mg/dL (0.010–0.052) (*p* = 0.02) (Figure 2). Additionally, excessive milk consumption exceeding the recommended daily amount was significantly associated with lower ascorbic acid levels (*p* = 0.033). However, there was no significant correlation between ascorbic acid level and gender or developmental status.

**Figure 3:**
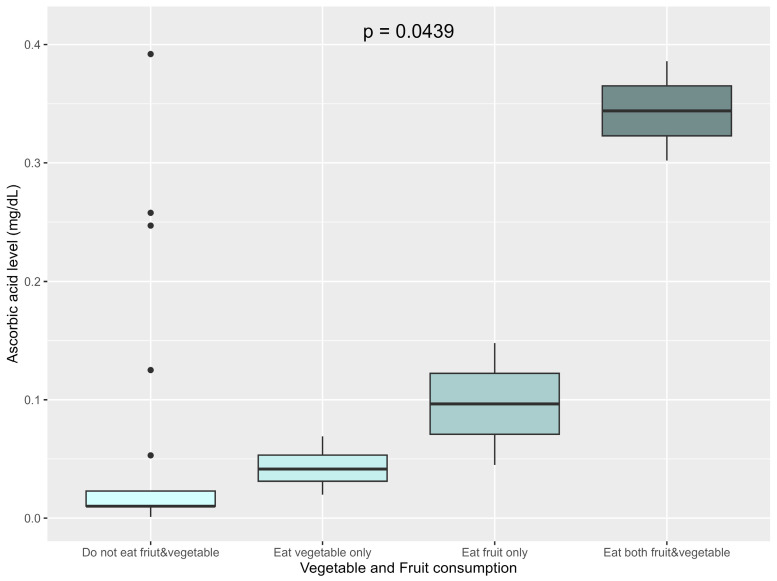
Association between vegetable and fruit consumption and ascorbic acid levels.

When focusing only on fruit and vegetable consumption as concerns serum ascorbic acid levels, the patients were classified into four groups: those who consumed both fruits and vegetables, those who only consumed vegetables, those consuming only fruits, and those who consumed neither. Serum ascorbic acid levels were significantly associated with fruit and vegetable consumption (*p* = 0.04). The highest levels were observed among those that consumed both fruits and vegetables (median 0.344 mg/dL, IQR 0.323–0.365), followed by those who consumed only fruits (median 0.097 mg/dL, IQR 0.07–0.122), and those that only consumed vegetables (median 0.042 mg/dL, IQR 0.031–0.043). The lowest levels were found in those having consumed neither fruits nor vegetables (median 0.010 mg/dL, IQR 0.010–0.023) (Figure 3).

### 3.7. Treatment and Treatment Outcomes

All patients diagnosed with vitamin C deficiency in this study were prescribed oral vitamin C supplementation. The mean (SD) loading dose was 292.7 (64.8) mg/day for the first week, followed by a maintenance dose of 108.9 (35.8) mg/day, for a total treatment duration of three months. In addition to vitamin C supplementation, individualized feeding therapy was provided to ensure adequate dietary intake of vitamin C rich foods, particularly vegetables and fruits. For patients that had difficulty consuming these, fresh fruit juice was recommended to support their vitamin C intake.

Among the 46 patients, 34 patients (73.9%) were treated as inpatients. All patients showed clinical improvement, with the mean time to clinical response after starting treatment being 3 days (IQR: 2–3.5): median duration of hospitalization was 8 days (IQR: 5–16.5). Among inpatient cases, 3 out of 30 patients (10%), who initially presented with refusal to walk and limb pain, achieved complete symptom resolution by the time of discharge.

The median follow-up time was 25 days (IQR: 17–365), and a total of 41 patients (89%) were followed up with after treatment. Of these, 26 patients (63.4%) reported resolution of initial symptoms, while the remaining 15 patients (36.6%) still had some mild residual symptoms.

Serum ascorbic acid levels were reassessed in 14 patients after one month of treatment. Among them, 12 patients (85%) reached normal ascorbic acid levels, while 2 patients (14.3%) had persistently low levels, despite full resolution of their clinical symptoms. At the time of follow-up, dietary changes were observed, with 18 patients (43.9%) reporting increased fruit and vegetable consumption. Fourteen patients (34.1%) reported consumption of fruit juices, and nine patients (21.9%) continued to avoid fruits and vegetables; although they remained on vitamin C supplementation.

## 4. Discussion

Although vitamin C deficiency is considered rare in high-income countries, it continues to be diagnosed with notable frequency in Southeast Asian countries, especially in Thailand. In this cohort, 46 patients were diagnosed over two decades. This is consistent with a previous study conducted in Bangkok, central Thailand in which high prevalence was reported: 106 cases were diagnosed by ascorbic acid levels or radiologic findings compatible with vitamin C deficiency within thirteen years [24]. Similarly, studies reported a male predominance and high prevalence among the preschool age group, likely due to selective eating behaviors that are well recognized during this developmental stage [33]. However, numerous cases of vitamin C deficiency have also been reported in adults [34,35], indicating that this condition can occur at any life stage.

More than half of the patients in this study had underlying medical conditions such as behavioral problems and neurological impairment, which is consistent with trends reported in the literature [4,11,36]. Iamopas et al. [24] reported that 44 out of 106 (41.5%) Thai children diagnosed with vitamin C deficiency also had cerebral palsy, epilepsy, or autism spectrum disorder, which is consistent with the comorbidities observed in this study. These comorbidities often worsen eating behaviors and increase vulnerability to micronutrient deficiencies including vitamin C [4,7,8,12,13,37]. However, vitamin C deficiency has also been reported in children without underlying medical conditions [22,24,38]. In this study, 40% of the patients had no underlying medical conditions. Moreover, more than half of the vitamin C deficiency patients classified as having a normal nutritional status were overweight. Again, this is consistent with a previous Thai study [24] in which 80% of vitamin C deficiency patients having normal nutritional status were overweight as well. This indicates that vitamin C deficiency can occur even in patients without malnutrition, if they have poor dietary habits. Without careful assessment of risk factors for vitamin C deficiency, under-recognition and delayed diagnosis may occur in this group.

Regarding dietary history, all patients exhibited inappropriate eating patterns. This was characterized by limited diet variety, insufficient intake, and inappropriate food texture, especially among patients with neurological impairments, which is likely attributable to oromotor dysfunction. Poor dietary habits were common across all groups, including low fruit and vegetable consumption, frequent intake of non-nutritive snacks, and excessive milk consumption: primarily UHT milk, which is known to lose vitamin C during processing [16]. This indicates that even children with normal development and no physical limitations are susceptible to unhealthy eating patterns that can lead to vitamin C deficiency as well as potentially other micronutrient deficiencies.

While previous Thai studies reported that up to 96% of vitamin C deficient children failed to consume sufficient fruits or vegetables [24], this cohort revealed a slightly lower prevalence, with 70% not consuming fruits nor vegetables. Importantly, among those that did consume fruits and vegetables, intake was irregular and quantities were inadequate. This confirms that both low quantity and inconsistent intake are sufficient to precipitate deficiency.

Serum ascorbic acid levels were significantly associated with fruit and vegetable intake; again affirming their role as primary vitamin C sources [1]. High intake of milk with high-temperature processing correlated with lower ascorbic acid levels, potentially because excessive milk consumption may reduce the intake of other nutrient-dense food [24]. Although, previous research has suggested that the male gender as a risk factor [10,39], this is likely due to greater, absolute lean body mass and hypothetical volumetric dilutional effects. In this study, males tended to have lower ascorbic acid concentration; however, the difference was not statistically significant. Although developmental delay has previously been suggested as an important risk factor [4,37], it was not significantly associated with ascorbic acid levels in this study. This may possibly be because both developmentally delayed and normal development patients had similarly low fruit and vegetable consumption, which is the key factor leading to vitamin C deficiency.

From a clinical perspective, manifestations paralleled those reported previously. Musculoskeletal symptoms predominated, with leg pain found in 92.5% of patients in the study by Iamopas et al. [24], similar to our finding of 76% with limb pain. Radiographic findings frequently showed osteopenia, a nonspecific but common sign [15,38]. The pathognomonic radiographic sign white line of Frankel was the most frequently observed, presenting in 47.6% of this cohort, compared to 74.5% reported previously in another Thai study [24]. However, the absence of radiological changes does not exclude deficiency, as changes typically arise after prolonged deficiency lasting 3–6 months [11,24,40].

Other micronutrient deficiencies were observed as a comorbidity. Iron deficiency was common, reflecting the role of vitamin C in enhancing non-heme iron absorption [11,41]. Deficiencies of vitamin D, zinc, vitamin A, and vitamin B1 were also observed and are likely related to the restrictive eating behaviors leading to multiple micronutrient insufficiencies. These results are consistent with Van Heerden et al.’s findings, wherein 70% of children with vitamin C deficiency had at least one other micronutrient deficiency [37]. This highlights the need for comprehensive nutritional assessments to guide appropriate management.

Treatment regimens in previous reports have varied in dosage and duration, ranging from 300 to 1000 mg/day, with consistently favorable outcomes [12,22,40]. In this study, patients were treated with oral vitamin C at a loading dose of 300 mg/day for one week, followed by a maintenance dose of 100 mg/day for three months; all patients demonstrated clinical improvement. One third of children, initially presenting with musculoskeletal symptoms, continued to have residual symptoms. This is consistent with the findings of Trapani et al. [11], who reported that 76% of cases achieved full recovery after an interval treatment period of 1–11 months. The persistence of symptoms in some cases was likely related to behavioral adaptation or fear of weight-bearing. Two patients in our study continued to have low serum ascorbic acid levels at follow up, despite resolution of clinical symptoms. The slow recovery of ascorbic acid levels was possibly related to continued avoidance of fruits and vegetables at follow-up, which are rich sources of vitamin C, and possibly due to poor adherence to supplementation. However, compliance data were unavailable because of the retrospective nature of this study.

Nearly half of the patients showed improvements in their dietary habits after receiving dietary counseling, including increased consumption of fruits and vegetables. The most commonly consumed fruits were oranges and guavas, both of which are good sources of vitamin C. Although previous evidence indicates that blackcurrants, kiwifruit, and various citrus fruits contain some of the highest levels of vitamin C [2,10], these foods are less accessible in Thailand. Although commonly available vegetables, such as kale, chili peppers, and broccoli, also provide substantial amounts of vitamin C [1,10], many children still had difficulty consuming adequate quantities of these vegetables. The observed improvements in dietary habits after counseling suggest that appropriate dietary counseling and parental education can promote healthy eating behaviors and may help prevent vitamin C deficiency.

The strength of this study is that the dataset covers a period of up to 20 years. Furthermore, this study included subgroup analysis by developmental status, which provided insights into differences in dietary patterns and identified opportunities for tailored nutritional interventions. However, this study has some limitations. First, this is a retrospective study, which has several limitations owing to its design such as missing data, incomplete ascorbic acid level, recall bias, and inconsistent documentation. Second, the data were obtained from patients at a single tertiary-level hospital center, and we were unable to accurately estimate the prevalence of vitamin C deficiency in our cohort because the study population did not encompass all pediatric patients during the study period. Lastly, socioeconomic status may be a contributing factor to vitamin C deficiency; however, our study had limited data.

This study highlights the critical need for clinicians to consider vitamin C deficiency in children, both for patients without underlying medical conditions or malnutrition. Early recognition of characteristic clinical and radiographic signs can facilitate prompt diagnosis and intervention. Subgroup analyses, according to developmental status and underlying disease, revealed targeted opportunities for personalized nutritional interventions. Detailed dietary history-taking and parental education on balanced nutrition is crucial to prevent vitamin C deficiency as well as other micronutrient insufficiencies. This can ultimately reduce disease burden and improve pediatric health outcomes.

## 5. Conclusions

Vitamin C deficiency remains an important but often under-recognized condition in children. In cases of unexplained limb pain, refusal to walk, or bleeding gum, especially among patients with neurodevelopment disorders or selective eating behaviors, vitamin C deficiency should be considered. Dietary assessment, focusing on both the quantity and quality of fruit and vegetable intake in all children, is essential to prevent misdiagnosis and delays in treatment. Strengthening nutritional education for parents and promoting healthy eating habits in early childhood are key strategies to reduce the incidence and long-term consequences of this preventable condition.

## Figures and Tables

**Table 1:** Baseline Characteristic and Demographic Data of Patients Diagnosed with Vitamin C Deficiency.

	Total (%) N = 46	Group			
		Normal Development (%) N = 21	Neurological Impairment (%) N = 14	Behavioral Problem (%) N = 11	*p*-Value
Age (y), median (IQR)	4.8 (2.6, 8.4)	2.9 (2.3, 5.9)	8.1 (5.7, 9.9)	3.8 (3.0, 7.8)	0.018
Age (y), No (%)					0.011
<5 years old	23 (50)	15 (71.4)	2 (14.3)	6 (54.5)	
5–10 years old	16 (34.8)	5 (23.8)	8 (57.1)	3 (27.3)	
>10 years old	7 (15.2)	1 (4.8)	4 (28.6)	2 (18.2)	
Gender, No (%)					0.241
Male	28 (60.9)	12 (57.1)	7 (50)	9 (81.8)	
Female	18 (39.1)	9 (39.1)	7 (50)	2 (18.2)	
	**N = 42**	**N = 21**	**N = 10**	**N = 11**	
Baseline nutritional status, No (%)					<0.001
Normal	23 (54.8)	16 (76.2)	0 (0)	7 (63.6)	
Wasting	13 (31.0)	2 (9.5)	9 (90.0)	2 (18.2)	
Overweight or obesity	6 (14.2)	3 (14.3)	1 (10.0)	2 (18.2)	

**Table 2:** Dietary history of patients diagnosed with vitamin C deficiency.

	Total (%) N = 46	Group			
		Normal Development (%) N = 21	Neurological Impairment (%) N = 14	Behavioral Problem (%) N = 11	*p*-Value
Inappropriate food texture for age	15 (32.6)	6 (28.6)	8 (57.1)	1 (9.1)	0.042
Inadequate consumption of rice (N = 45)	36 (80)	18 (85.7)	13 (92.9)	5 (50.0)	0.037
Inadequate consumption of meat (N = 42)	31 (73.8)	17 (89.5)	9 (75)	5 (45.4)	0.04
No consumption of vegetables (N = 43)	33 (76.7)	16 (84.2)	8 (57.1)	9 (90.0)	0.139
Frequency of vegetable consumption (days/week)	3.2 (2.6, 6.1) ^1^	4.8 (3.6, 5.9) ^1^	3.5 (3.2, 5.2) ^1^	1.5 (1.5, 1.5) ^1^	NA
No consumption of fruits (N = 45)	33 (73.3)	14 (66.7)	10 (76.9)	9 (81.8)	0.687
Frequency of fruit consumption (days/week)	2.2 (1.9, 3.3) ^1^	3 (2.5, 4) ^1^	2 (2, 2) ^1^	1.2 (1.1, 1.4) ^1^	NA
Consumption of non-nutritive snack (N = 21)	18 (85.7)	7 (100)	6 (85.7)	5 (71.4)	0.742
Consumption of juice (N = 15)	5 (33.3)	4 (57.1)	0 (0)	1 (25.0)	0.254
Consumption of milk	38 (82.6)	18 (85.7)	12 (85.7)	8 (72.7)	0.613
Median of milk consumption (mL/day)	1040 (360, 1619) ^1^	1225 (520, 1812.5) ^1^	490 (350, 927.5) ^1^	1012.5 (592.5, 1350) ^1^	0.304
Median of milk consumption relative to recommendation	2.6 (0.9, 4) ^1^	3.1 (1.3, 4.5) ^1^	1.2 (0.9, 2.3) ^1^	2.5 (1.5, 3.2) ^1^	0.297

^1^ Median (IQR).

## Data Availability

The data presented in this study are available upon reasonable request from the corresponding author. The data are not publicly available due to privacy and ethical restrictions related to the protection of participants information.
